# Coherent ultrafast photoemission from a single quantized state of a one-dimensional emitter

**DOI:** 10.1126/sciadv.adf4170

**Published:** 2023-10-12

**Authors:** Chi Li, Mengxue Guan, Hao Hong, Ke Chen, Xiaowei Wang, He Ma, Aiwei Wang, Zhenjun Li, Hai Hu, Jianfeng Xiao, Jiayu Dai, Xiangang Wan, Kaihui Liu, Sheng Meng, Qing Dai

**Affiliations:** ^1^ CAS Key Laboratory of Nanophotonic Materials and Devices, National Center for Nanoscience and Technology, Beijing 100190, China.; ^2^ Beijing National Laboratory for Condensed Matter Physics and Institute of Physics, Chinese Academy of Science, Beijing 100190, China.; ^3^ Centre for Quantum Physics, Key Laboratory of Advanced Optoelectronic Quantum Architecture and Measurement (Ministry of Education), School of Physics, Beijing Institute of Technology, Beijing 100081, China.; ^4^ State Key Laboratory for Mesoscopic Physics, Frontiers Science Centre for Nano-optoelectronics, School of Physics, Peking University, Beijing 100871, China.; ^5^ Department of Physics, Hunan Key Laboratory of Extreme Matter and Applications, National University of Defense Technology, Changsha 410073, China.; ^6^ National Laboratory of Solid State Microstructures and School of Physics, Collaborative Innovation Center of Advanced Microstructures, Nanjing University, Nanjing 210093, China.

## Abstract

Femtosecond laser–driven photoemission source provides an unprecedented femtosecond-resolved electron probe not only for atomic-scale ultrafast characterization but also for free-electron radiation sources. However, for conventional metallic electron source, intense lasers may induce a considerable broadening of emitting energy level, which results in large energy spread (>600 milli–electron volts) and thus limits the spatiotemporal resolution of electron probe. Here, we demonstrate the coherent ultrafast photoemission from a single quantized energy level of a carbon nanotube. Its one-dimensional body can provide a sharp quantized electronic excited state, while its zero-dimensional tip can provide a quantized energy level act as a narrow photoemission channel. Coherent resonant tunneling electron emission is evidenced by a negative differential resistance effect and a field-driven Stark splitting effect. The estimated energy spread is ~57 milli–electron volts, which suggests that the proposed carbon nanotube electron source may promote electron probe simultaneously with subangstrom spatial resolution and femtosecond temporal resolution.

## Teaser

Demonstrating coherent ultrafast photoemission from carbon nanotubes, which may promote the resolution of electron pulses.

## INTRODUCTION

Femtosecond-resolved electron probe is the fundamental tool to characterize atomic-scale ultrafast dynamics, as well as the key component of various free-electron radiation sources (*1*–*6*). However, the energy spread of the photoemission is the key limiting factor of spatial and temporal resolution of the electron probe, as well as the energy resolution of ultrafast electron-energy loss spectroscopy (*7*). Normally, the energy spread of <100 meV is required to achieve subangstrom spatial resolution and femtosecond temporal resolution simultaneously, but intense laser excitation of metallic nanotips may induce large ones (>600 meV) (*8*–*15*) due to the emitting energy level broadening effect (*16*–*18*). A monochromator module can efficiently reduce the energy spread, but it will filter out ~99% of the beam current as a trade-off (*19*, *20*). Therefore, it is not applicable to the ultrafast electron sources, as their beam current (<1 pA) (*5*) cannot be cut down anymore to keep an acceptable single-noise ratio.

Therefore, effective approaches are proposed to reduce the energy spread of the ultrafast electron source, such as introducing quantum resonant tunneling effect (*14*, *21*, *22*). Resonant tunneling occurs in systems where a quantum well is sandwiched between two potential barriers (i.e., double-barrier structure). The electronic wave resonance at a narrow quantized energy level in the quantum well results in an intense and sharp electron emission peak (*23*), which generates electron beam with both high brightness and high monochromaticity (*19*). On the basis of this principle, serving as a quantum well, a zero-dimensional (0D) quantum dot has been proposed to be mounted on top of a metallic nanotip (i.e., electron reservoir) (*24*, *25*), which leads to a reduced energy spread of ~300 meV (*25*). However, the electron emission may be contributed by multiple energy levels in the quantum dot, as the energy distribution of the laser-excited electrons (>1 eV) in the metallic nanotip is usually one order of magnitude broader than the gap of the energy levels (<100 meV) in the quantum dot (*26*).

To further reduce the energy spread, the double-barrier structure should be optimized as following. First, the number of energy levels in the quantum well should be limited to ensure that the emission is mainly contributed by an individual energy level. Second, the electron reservoir should provide a single sharp excited state, which further reduces the energy spread of the resonant tunneling field emission. Third, the potential barrier between the quantum well and the reservoir should be adjustable to precisely control the emission efficiency. Obviously, these optimizations are hard to be fully achieved in metal nanostructures but were believed to be tailor-made for carbon nanotubes (CNTs) with a 1D body and a 0D cap (*27*–*30*). However, the theoretical demonstration and the experimental construction of double-barrier structure on a CNT tip have not been realized.

Here, we demonstrate the constructing of a double-barrier structure on a CNT tip for resonant tunneling–type ultrafast photoemission. By tuning the electronic structure through the thermal control of the carrier concentration, precise control of the electron emission through an individual energy level was realized. The resonant tunneling was evidenced by a laser-induced negative differential resistance (NDR) effect (*31*, *32*), which suggests that a photoemission occurs when the 1D excited state in the CNT body is aligned with an individual quantized energy level confined within the 0D quantum well (*33*–*38*). The quantized energy level in 0D cap can be controlled by adjusting the static voltage. As the static voltage increases, the original alignment of 1D excited state in the CNT body with the quantized energy level in 0D cap becomes mismatched, the photoemission current decreases, and the NDR peak appears. In addition, a splitting of the NDR peak was identified, which is attributed to the Stark splitting (*39*, *40*) of two degenerate quantized states induced by combined effect of the static field and the laser field. This suggests that the quantized energy levels can be further finely tuned to achieve a more controllable electron emission. The present findings are relevant for the design of future ultrafast electron sources (*41*, *42*).

## RESULTS

**Fig. 1.**
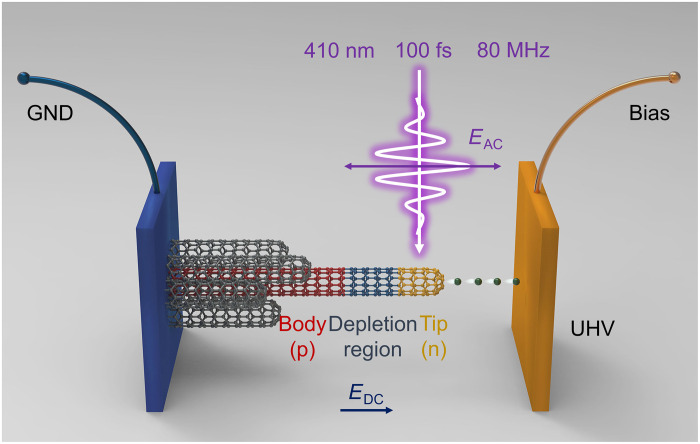
Overview of the experimental setup. The CNT electron emitter is driven by a static bias voltage (which induces a static field *E*_DC_) and a 100-fs laser pulse (optical field, *E*_AC_) with a central wavelength of 400 nm. A p-n junction is formed at the tip, induced by *E*_DC_. All experiments were carried out in an ultrahigh vacuum (UHV) chamber at cryogenic temperatures.

Figure 1 shows the experimental setup (see the Supplementary Materials and fig. S9 for details) (*33*, *36*). Combined with a static bias voltage (*V*), 100-fs-width laser pulses with a central wavelength of 400 nm and a repetition rate of 80 MHz were used to drive the electron emission. The laser was linearly polarized and was normally incident on top of the CNT cluster emitter. Both the static field (*E*_DC_) and laser field (*E*_AC_) were parallel to the CNT axis. The focus spot was 10 μm in diameter. The distance between anode and cathode was adjusted using a mica spacer to be ~100 μm. Thus, the macro static field is 0.5 V/μm at a bias voltage of 50 V. Given that the static field enhancement factor is 3000 (*43*), the average value of the enhanced static field at the CNTs tips was estimated to be ~1.5 V/nm.

Because of the p-type semiconducting nature of as-grown single-wall CNTs (*44*), when a bias voltage is applied, a nanoscale inverse layer (n-type) is induced at the tip, which is a 0D quantum well (*45*). A nanoscale depletion region is then formed between the tip and the body, which can function as a tunneling barrier (*46*). Therefore, a double-barrier tunneling structure is formed at the emitting tip (*47*, *48*). The width of the depletion region, which is determined by its capacitance (*C*_d_), is largely dependent on the carrier concentration of the entire CNT (*N*_c_) (*49*). Through Coulomb blockade, the electronic structure of the quantum well is renormalized (*50*) into a series of equidistant energy levels with an energy gap of Δ*E* = *e*^2^/*C*_d_, where *e* is the charge of a single electron and *C*_d_ = ɛ*S*/*D*, where *D* is the width of the depletion region, *S* is the cross-sectional area of CNT, and ɛ is the dielectric constant of CNT. Therefore, the energy gap of the quantum well can be tuned by reducing the carrier concentration (*N*_c_) at cryogenic temperatures.

**Fig. 2.**
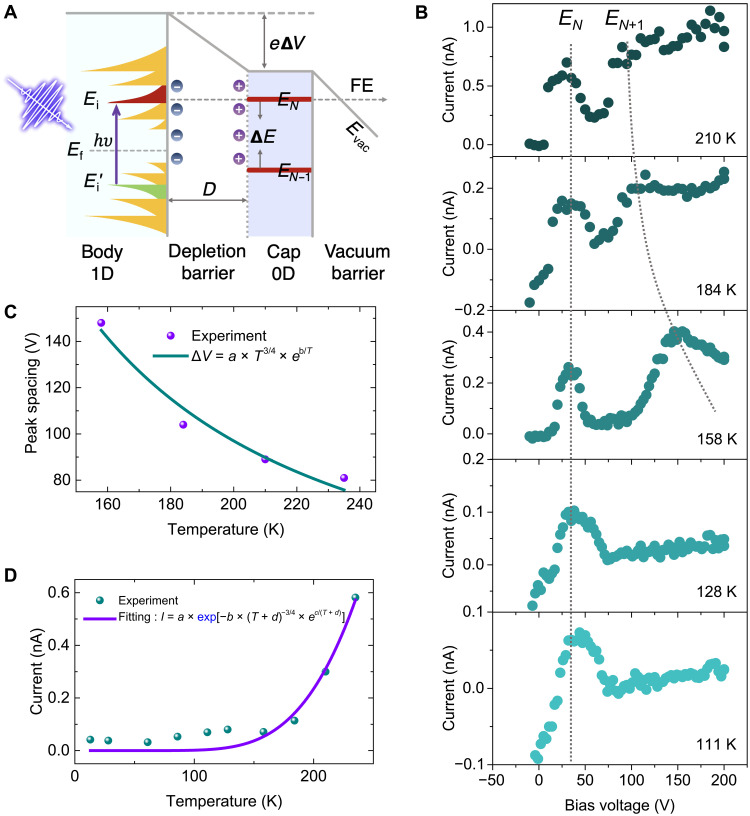
Laser-induced NDR effect. (**A**) Diagram of the ultrafast photoemission process. Electrons are excited from an energy level (*E*_i_′) in the valence band to a corresponding energy level (*E*_i_) in the conduction band. (**B**) *I*-*V* curves at different temperatures ranging from 111 to 210 K. All the curves were obtained at a laser power of 3 mW. The dotted lines mark the positions of the two peaks, which indicate that the peak spacing decreases as the temperature increases. (**C**) Experimentally obtained voltage spacing of two adjacent NDR peaks as a function of temperature. The green curve is the corresponding fitting. (**D**) Experimentally obtained dependence of the peak current of the first NDR peak on temperature. The purple curve is the corresponding fitting.

On the basis of the above design, the band diagram of the CNT emitting tip and the corresponding laser-assisted resonant tunneling field emission process can be depicted as shown in Fig. 2A. The CNT body has a 1D electronic structure, while the tip has a 0D electronic structure. First, the electrons are excited into a van Hove singularity (VHS) (e.g., the *E*_i_ level shown in Fig. 2A) in the conduction band from the corresponding VHS (*E*_i_′) in the valence band. Second, the excited electrons are field-emitted through a quantized energy level (*E_N_*) in the quantum well in the resonant tunneling regime as *E_N_* can be aligned with the VHS by applying a suitable bias voltage (*51*). On the basis of this model, a repeatable and tunable NDR effect (*52*) should be observed, as it will be shown below. However, in this regime, electron emissions from multiple energy levels may be superimposed together because of the alignment of different energy levels in the quantum well to different excited states in the CNT body. To achieve a highly controllable electron emission from an individual quantized state, both the energy gap of the excited states in the 1D body and the Coulomb-blockade energy gap (Δ*E*) in the 0D tip should be fully expanded.

To increase the energy gap of the excited states, laser pulses with a high photon energy should be used. To increase Δ*E*, the width of the depletion region should be increased by decreasing the carrier concentration at lower temperatures. Consequently, we chose femtosecond laser pulses with a photon energy of 3.1 eV (and a central wavelength of 400 nm) to drive the ultrafast photoemission at cryogenic temperatures. Multiple NDR peaks are clearly observed in Fig. 2B at temperatures above 210 K, while the NDR peak spacing exhibits a pronounced temperature dependence—it decreases with increasing temperature. This suggests that the appearance of the two peaks is due to the alignment of an individual excited state (*E*_i_) to neighboring discrete levels (*E_N_* and *E*_*N*+1_; *E*_*N*+1_ is not shown) in the quantum well driven by the bias voltage. Here, the peak spacing is linearly proportional to Δ*E*. To support this conclusion, detailed data fittings were carried out as follows. First, in intrinsic region, the carrier concentration (*N*_c_) is exponentially proportional to the temperature (*T*) — $N_{c}\propto T^{\frac{3}{2}}e^{-\frac{1}{T}}$. The width of the depletion region is then calculated as $D\propto \sqrt{1/N_{c}}\propto T^{-\frac{3}{4}}e^{\frac{1}{T}}$, the capacitance of the barrier is *C*_d_ ∝ 1/*D*, and the NDR peak spacing is $\text{Δ}V\propto \text{Δ}E\propto \frac{1}{C_{d}}\propto T^{-\frac{3}{4}}e^{\frac{1}{T}}$. Using this relationship, the dependence of the NDR peak spacing on temperature could be well fitted, as shown in Fig. 2C. Second, in the double-barrier structure, as a strong static field (~1.5 V/nm) is applied to the CNT tip, the vacuum barrier is considerably more transparent to the excited states than the depletion barrier (*16*). Thus, at a fixed *E*_DC_, the field emission current (*I*) can be effectively modulated by tuning the width of the depletion barrier. Therefore, $I\propto e^{-D}\propto e^{\left(T^{-\frac{3}{4}}e^{\frac{1}{T}}\right)}$. Using this relationship, the dependence of *I* on temperature could also be well fitted when the temperature is higher than 160 K, as shown in Fig. 2D. Below 160 K, the data points deviated from the fitting, which indicates the carrier concentration-temperature relationship, are in extrinsic region. It can thus be concluded that ultrafast photoemission from a single quantized energy level can be achieved using the Coulomb blockade effect at cryogenic temperatures and driving laser pulses with a photon energy of 3.1 eV. The result is reproducible, and another group of temperature-dependent data is shown in fig. S12. Note that, with the laser intensity increases, more VHS should be filled with excited electrons, which results in additional NDR peaks as shown in figs. S2 and S3.

**Fig. 3.**
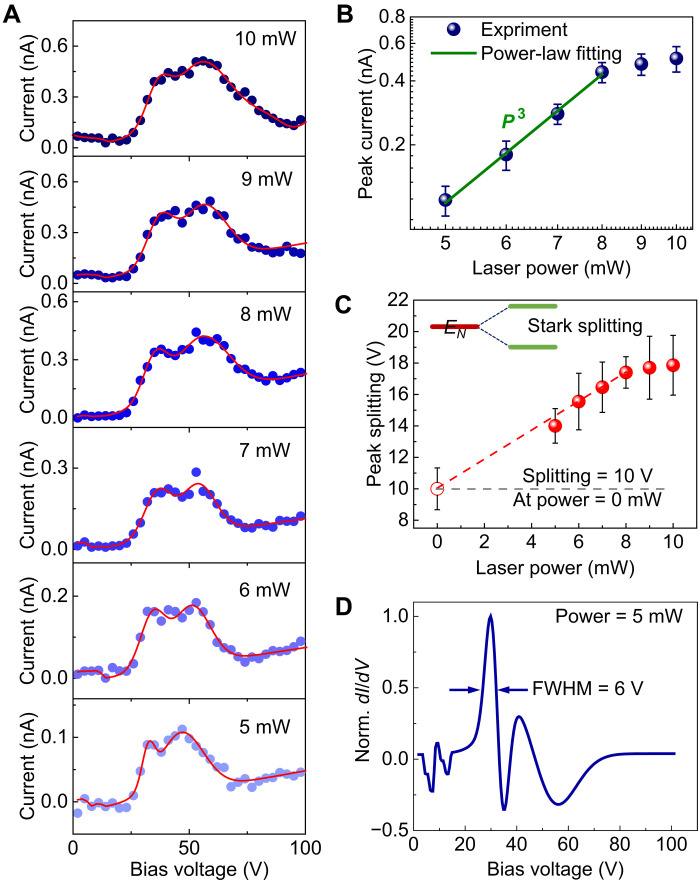
Single-electron emission and laser field–induced Stark splitting. (**A**) *I*-*V* curves obtained at different powers ranging from 5 to 10 mW; the corresponding peak fittings are shown by the red curves. All curves were obtained at 20 K. (**B**) Emission current as a function of the laser power at a bias voltage of 50 V. (**C**) Experimentally obtained splitting voltage as a function of the laser power. The hollow circle represents the estimated splitting voltage at 0 mW power based on experimental data (solid circle). The inset shows the diagram of the Stark splitting effect (the two green lines) of an energy level (*E_N_* in Fig. 2A). (**D**) *dI*/*dV* of the data in (A) at 5 mW, which indicates a rising edge width of ~6 V.

**Fig. 4.**
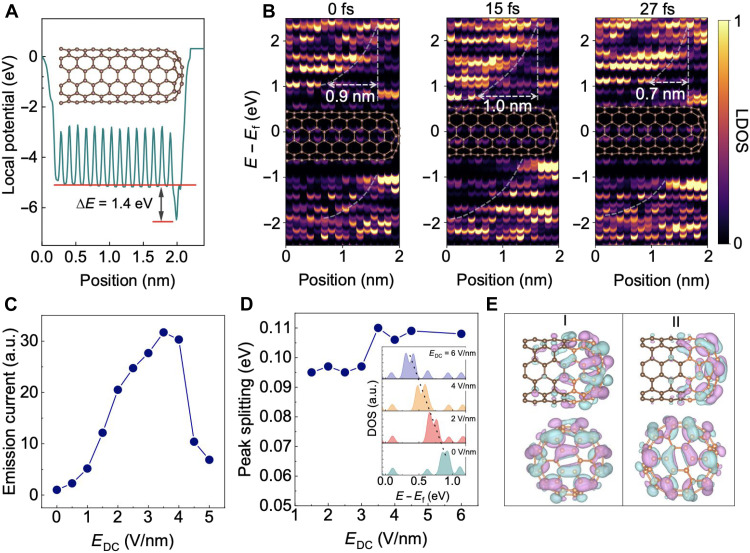
Theoretical overview of the coherent emission process. (**A**) Ground-state electrostatic potential along the axial direction of the CNT. The inset shows the atomic structure of the CNT. (**B**) LDOS of the CNT at different times during a 30-fs laser pulse. The LDOS is averaged by summing carbon atoms with the same position along the CNT axis. Here, the laser pulse reaches its maximum optical field strength of 2 V/nm at 15 fs and ends after 27 fs. The width of the depletion potential barrier varies with the intensity of the laser. (**C**) Dependence of the current *I* on the amplitude of the static electric field *E*_DC_; here, the waveform of the laser pulse is fixed. a.u., arbitrary units. (**D**) Simulated splitting energy as a function of *E*_DC_. The inset shows the atom-resolved partial DOS at the tip of the CNT and its evolution under different static electric fields. (**E**) Calculated isosurfaces of the wave functions in the real space for the two split peaks shown in (D): The two top images show the side view, while the two bottom ones show the top view. The carbon atoms that make up the nanotube body and tip are marked by orange and brown colors, respectively.

Since the energy levels are renormalized by Coulomb blockade, the ultrafast photoemission should be in the single-electron regime. To further investigate the details of this unique emission mechanism, the *I*-*V* curves were measured for different powers at the much lower temperature of ~20 K, as shown in Fig. 3A. The photoemission current–laser power (*I*-*P*) curve shows nonlinear photoemission (third power law) behavior (Fig. 3B), which is consistent with the polarization-dependent photoemission current curve shown in fig. S11. It is noticed that a saturation in the peak current occurs at 8 mW, as shown in Fig. 3B. The saturation can be attributed to single-electron emission because of Coulomb blockade. In this regime, two parameters, namely, the time spacing of the periodical single-electron emission (τ) and the lifetime of the excited electrons (Τ), determine the upper-limit number of the emitted electrons (*m*) in one laser pulse. *m* can be roughly estimated as $m=\frac{T+T_{p}}{\tau }$, where *T*_p_ is the width of the laser pulse (100 fs). Therefore, the photoemission can be divided into two stages. In the first stage (below saturation, i.e., when the power is less than 8 mW), the emission current is not limited by the lifetime of the excited states, and it therefore behaves as a photoemission current obeying a nonlinear power-law scaling. In the second stage (when the power is greater than 8 mW), *m* reaches the saturation limit, which leads to a deviation in the *I*-*P* curve from the power-law scaling. Note that the observed third-order power-law scaling may not directly correspond to a simple multiphoton absorption (*53*, *54*) because of several factors such as electron-electron interaction (*55*), variable depletion barrier size (see Fig. 4B), and heating effect (see Fig. 2A) induced by laser illumination. First, the *e*-*e* interaction induces a notable Auger recombination process, which increases the order of multiphoton photons. Second, the width of the depletion potential barrier varies with the intensity of the laser, potentially affecting the photoemission behavior. In addition, nonequilibrium hot electrons induced by laser illumination can also contribute to higher-order multiphoton photoemission. Figure S8 displays the simulated photoemission current (*I*) as a function of the laser power at a fixed static field (*E*_DC_). It is clear that the scaling varies with the laser field (*E*_AC_) and static field, which suggests that it cannot be evaluated by a simple multiphoton absorption regime.

By further inspecting the NDR peaks in Fig. 3A, a clear splitting is noticed. This is attributed to the Stark splitting (*39*, *40*) of the quantized energy level (the *E_N_* level in Fig. 2A) in the quantum well into two levels (as shown in the inset of Fig. 3C) modulated by the external electric field (both *E*_DC_ and *E*_AC_). The width of the peak splitting increases with increasing laser power and reaches saturation when the laser power exceeds 8 mW, as shown in Fig. 3C. The details of the splitting behavior and an evaluation of the photoemission energy level width will be provided in the following together with ab initio simulations based on the density functional theory (DFT) and the time-dependent density functional theory (TDDFT).

The diameter of the simulated CNT with a chiral index of (6,6) is ~1 nm, which is within the range of tube diameters used in the experiments (fig. S1B). The calculated electrostatic potential of the CNT is shown in Fig. 4A. A potential well is noticed at the tip (*56*). The averaged local potentials near the CNT body and cap are −5.1 and −6.5 eV, respectively, leading to an internal potential barrier with a height of 1.4 eV. To estimate the size of the depletion layer between the CNT body and tip, the space-resolved local density of states (LDOS) along the axial direction of the nanotube is shown in Fig. 4B. Here, the color map indicates the proportion of states that are to be occupied by the electrons; that is, the larger the LDOS, the larger the electronic potential energy at each energy. It is clear that a heterogeneous DOS distribution is generated in such a quantum confined structure, and the triangular potential barrier bottom width and height are 0.9 nm and 1.4 eV, respectively. Under laser illumination, the depletion layer is deformed because of the carrier excitation, while the potential barrier still exists albeit with a modified transient barrier size.

The simulated dependence of the photoemission current (*I*) on *E*_DC_ at a fixed waveform of the laser pulse (fig. S5) is displayed in Fig. 4C. *I* increases with the increase in *E*_DC_ when *E*_DC_ < 3.5 V/nm and is sharply suppressed when *E*_DC_ = 5 V/nm, which demonstrates the occurrence of the NDR effect. The energy distribution of the excited carriers at different *E*_DC_ values is shown in fig. S6. In the range of 0 < *E*_DC_ < 4 V/nm, most electrons are excited from the discrete states with an energy of around −1.3 eV; the increased *E*_DC_ promotes the transition to the conduction band, lastly contributing to the emission into the vacuum. When *E*_DC_ = 5 V/nm, the electronic excitation from the states with energy of around −1.3 eV is suppressed, leading to the peak shifting toward higher valence bands and a decrease in the emission current. The above discussion proves that the bias voltage can modulate the energy level matching between the CNT body and tip. On the basis of the optical selection rules, it is expected that the carrier transition probability will change depending on the symmetry and energy difference between the initial and final states. Therefore, the NDR effect can be attributed to the transition suppression between the unmatched states induced by the bias voltage.

Figure 4D shows the simulation results of the *E*_DC_-induced Stark splitting. Driven by the static electric field, the states (in the 0D tip) near the Fermi level shift toward a lower energy level (as shown in the inset of Fig. 4D and fig. S4). In addition, the previously degenerate states split into two energy levels with the peak splitting increasing with the strength of the field, lastly reaching saturation (Fig. 4D). Since the wave functions of the two states are distributed along the in-plane zigzag and armchair directions around the nanotip, respectively, their dipole moments are distinct and will acquire distinguishable energies under the external field (as shown in Fig. 4E). This type of degenerate state generally exists in highly symmetrical atomic structures, such as the hexagonal lattice of graphene.

The energy spread can be estimated by combining the experimental and simulation results of the Stark splitting. In the present work, both *E*_AC_ and *E*_DC_ may induce a Stark effect. For the splitting induced by the AC light field, the amplitude scales linearly with the laser power (*57*). Therefore, we can linearly extrapolate the fitting curve of the experimental data in Fig. 3C, and it is found that the splitting value is 10 V at a laser power of 0 mW. This suggests that the splitting voltage, purely induced by the *E*_DC_, is 10 V (±1.3 V). As shown in Fig. 3A, the experimental splitting occurs at an applied voltage with an upper limit of ~50 V (corresponds to an enhanced static field of 1.5 V/nm). Thus, according to the simulated data in Fig. 4D, at *E*_DC_ = 1.5 V/nm, the splitting energy is ~95 meV, which corresponds to the experimental splitting voltage of 10 V. Therefore, the width of the rising edge of the NDR peak, which represents the emitting energy level width, is ~6 V (for the 5-mW case, as shown Fig. 3D), corresponding to ~57 meV (±7 meV). Given this value, we can estimate the tunneling time (*t*) of a single electron using the formula: t=ℏ/Γ, where ℏ is reduced Planck constant and Γ is the energy level width. Thus, the estimated tunneling time is 10 fs, and the total emission time for eight electrons (5 mW case of Fig. 3A) is around 70 fs. In addition, the estimated energy level spacing (∆*E*) is ~1400 meV at 158 K (~150-V voltage spacing; Fig. 2B). Then, *C*_d_ can be estimated to be ~1.1 × 10^−19^ F, by using the equation *C*_d_ = *e*^2^/∆*E*_c_, where *e* is the electron charge. Note that this value (~57 meV) is the upper limit of the realistic 0D quantized energy level width (*E*_0_; see fig. S10 for details). The estimated energy spread does not include the part induced by the space-charge effect during electron propagation. However, this effect can be greatly reduced in the unique CNT emitter as it works in single-electron emission regime.

## DISCUSSION

Furthermore, the emission current is ~100 pA, which corresponds to eight electrons per pulse as the laser repetition rate is 80 MHz. The beam current and energy width are comparable with those of a continuous electron gun equipped with a state-of-the-art electromagnetic monochromator (*19*) and outperform the state-of-the-art ultrafast electron sources by orders of magnitude. As the electron emission is in the Coulomb blockade regime, the duration of the electron pulses can be freely modulated by the laser pulse width and intensity. For illustration purposes, we demonstrate the time-resolved dynamics of the photoemission in fig. S7, which shows that the duration of the photoemission is strongly correlated with the duration of the laser pulse.

In conclusion, both TDDFT simulation and femtosecond laser–driven photoemission experimental have revealed that quantized double-barrier structure can be precisely constructed in situ on a single CNT tip due to the strong Coulomb interaction. As a result, coherent ultrafast photoemission with an estimated emitting energy level width of ~57 meV is achieved. In future, the double-barrier structure can be optimized using atom-manufacturing technology, which can provide an enhanced beam current for various application occasions. In the meantime, using shorter or lower intensity laser pulses, the temporal width of the electron pulses can be further compressed that may approach the time-energy uncertainty limit. The excellent properties will be of great interest for a transform-limited electron microscopy with a subangstrom imaging resolution and a femtosecond temporal resolution.

## MATERIALS AND METHODS

### Growth and characterization of the CNT emitters

Vertically aligned single-walled CNT cluster arrays were grown on highly doped n-type silicon chips using chemical vapor deposition using an Al (10 nm) /Fe (1 nm) multilayer catalyst at a temperature of 900°C and a pressure of 10^−2^ mbar. Gaseous ammonia was used to etch the catalyst into nanoislands for the nanotube self-assembly process. Acetylene was used as the carbon feedstock. The growth process lasted for 30 s, resulting in 10-μm-tall CNT clusters. Before the photoemission experiments, the CNT emitters were annealed in hydrogen at 1000°C for 2 hours to remove impurities and adsorbates on the surface. The scanning electron microscopy image of the as-grown CNT cluster array is shown in fig. S1A. The diameters (*d*) of the CNTs were assessed from their radial breathing mode frequencies [ω_RBM_ = 248/*d* (cm^−1^ nm^−1^)] through Raman spectroscopy, as shown in fig. S1B.

### Ultrafast photoemission measurements

The experiments were carried out using a Ti:sapphire ultrafast laser (Spectra-Physics, Mai Tai-Series, SHG) with a 100-fs pulse width and an 80-MHz repetition rate. The spectrum of the 410-nm laser is measured as shown in fig. S13A, while the pulse duration is measured by a commercial autocorrelator as shown in fig. S13B. The laser was linearly polarized and was perpendicularly incident on top of the CNT cluster emitter. The focus spot was 10 μm in diameter. The CNT was precisely moved on the piezo stage (in steps of 10 nm) to find the position with the maximum emission current to ensure that the CNT emitter was in focus. Although the clusters contain many nanotubes, the growth kinetics were such that only a few individual tubes protruded repeatedly between growths from these clusters producing a nanoscopic apex. The NDR peak is a strong evidence of resonant tunneling emission from an individual quantized state of a single CNT emitter. All the experiments were conducted in a high-vacuum chamber (10^−7^ torr) at a cryogenic temperature. The anode electrode was isolated from the cathode using a thick mica insulating spacer. A Keithley 2400 source measurement unit was used to bias the anode with voltages of up to 200 V. The current data were tested separately with (*I*_photoemission_) and without (*I*_background_) laser excitation, as shown in fig. S14. The emission current data (*I*) are the photocurrent data (*I*_photoemission_) minus background current (*I*_background_)—*I* = *I*_photoemission_ − *I*_background_. To further illustrate the single-nanotube emission in relative low bias voltage, we carried out a point electron source projection microscopy (PPM) using a CNT cluster as the electron source as illustrated in fig. S15.

### Simulation model of the SWCNTs

In this work, we considered single-wall (6,6) nanotubes with one capped end. Because of the absence of periodic boundary conditions in the molecular calculations, it is necessary to saturate the carbon dangling bonds with hydrogen atoms, yielding a C_192_H_12_ tube with a diameter of 0.81 nm. Ground-state DFT simulations were performed with the Vienna ab initio simulation package (*58*) using a projector-augmented wave pseudopotential in conjunction with the Perdew-Burke-Ernzerhof functional (*59*). The energy cutoff of the plane-wave basis set was 400 eV, and only the Γ point was used to sample the Brillouin zone. The atomic structure of the tube was positioned in a cubic supercell with vacuum regions larger than 15 Å along the three directions and was fully relaxed until the force on each atom was less than 0.01 eV/Å. We calculated the electrostatic potential inside the cell and averaged macroscopically along the axial direction of the CNT. The constant electric fields with different amplitudes were applied parallel to the tube axis to investigate the evolution of the position-dependent LDOS.

### Carrier excitation dynamics under external fields

The excitation dynamics was monitored by tracking the changes in the number of electrons in different energy levels. This simulation was performed using the time-dependent ab initio package as implemented in SIESTA (*60*–*62*). To understand the electron behavior under different bias voltages, we projected the time-evolved wave functions [∣ϕ*_n_*(*t*)⟩] on the basis of the ground-state Kohn-Sham orbitals (∣ψ*_m_*⟩), where *n* and *m* are the state indices that denote increasing orbital energy. The number of excited electrons is defined as

$$\text{Δ}n(t)=\sum_{m}^{\mathrm{occ}}\sum_{n}^{\mathrm{unocc}}{|\langle \psi_{m}|S|\varphi_{n}(t)\rangle |}^{2}$$

where *S* is the overlap matrix expressed by numerical atomic-centered orbitals and the sum is over all the unoccupied electron states. The real-time trajectories of the excited states were obtained from simulations at intervals of 0.05 fs with the many-electron density self-consistently propagating, offering a direct microscopic insight into the ultrafast dynamics of electrons upon photoexcitation.
