# Supplementary material for: Coherent ultrafast photoemission from a single quantized state of a one-dimensional emitter
Source: Sci Adv. 2023 Oct 12;9(41):eadf4170. doi: 10.1126/sciadv.adf4170 (PMC10569710; doi:10.1126/sciadv.adf4170)
Supplement: Supplementary file 1 — Figs. S1 to S15 Legend for data file S1 [file sciadv.adf4170_sm.pdf]

Science Advances
AAAS

Supplementary Materials for

Coherent ultrafast photoemission from a single quantized state of a one-dimensional emitter

Chi Li *et al.*

Corresponding author: Qing Dai, daiq@nanoctr.cn; Sheng Meng, smeng@iphy.ac.cn;
Kaihui Liu, khliu@pku.edu.cn; Xiangang Wan, xgwan@nju.edu.cn

Sci. Adv. **9**, eadf4170 (2023)
DOI: 10.1126/sciadv.adf4170

The PDF file includes:

Figs. S1 to S15
Legend for data file S1

Other Supplementary Material for this manuscript includes the following:

Data file S1

Supplement Materials

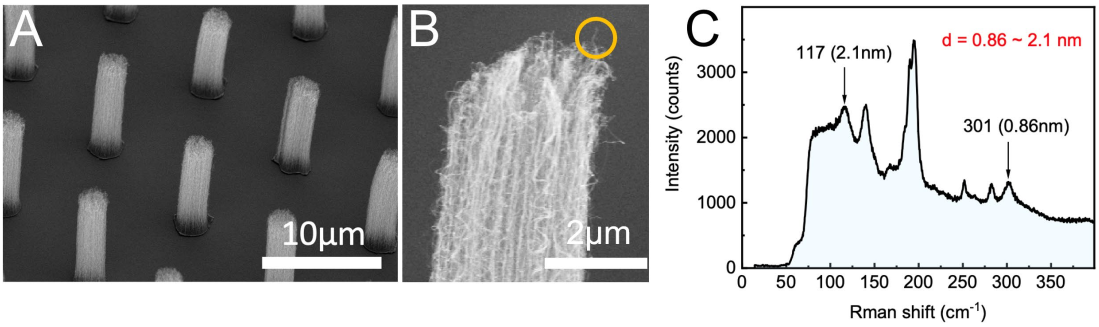

Fig. S1. Characterization of CNTs. (**A**) Scanning electron microscopy image of the as-grown CNT cluster array. (**B**) High magnification SEM images of a typical CNT cluster. This yellow circle is only intended to indicate that electron emission may come from some nanotubes with higher field enhancement factors that protrude from the cluster. (**C**) Raman spectrum of the CNT cluster, indicating diameters ranging from 0.86 to 2.1 nm.

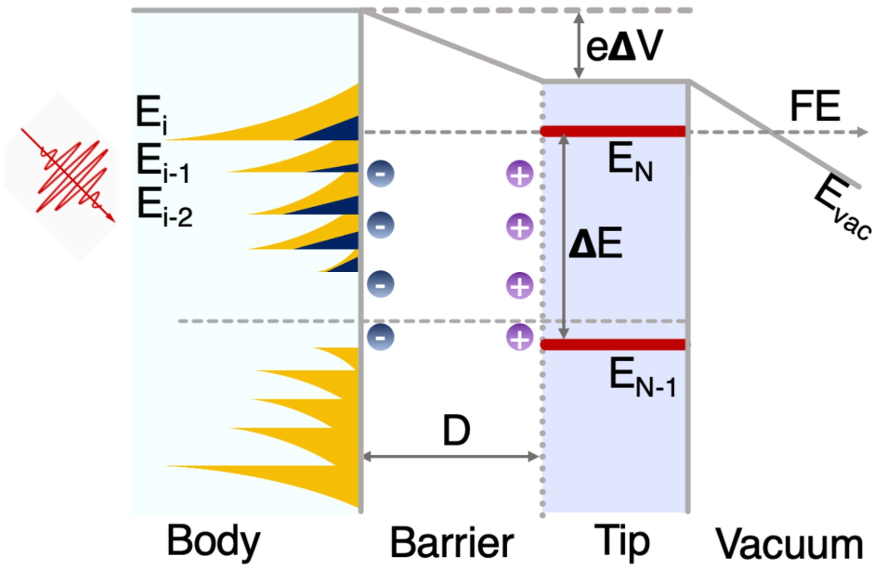

Fig. S2. Diagram of the ultrafast photoemission process at relatively high laser power and low temperature. By scanning the bias voltage, the quantized energy level (E_N) may be aligned to other VHS in the tube body—illustrated by E_i, E_{i-1}, E_{i-2}. It should be noted that, as the laser intensity increasing, more VHS should be filled with excited electrons, which results in additional NDR peaks. Consistent with this speculation, we observe multipeak in 0-200V bias range when the laser power is higher than 5 mW, as shown in Fig. S3. This further evidences the proposed model in the present work.

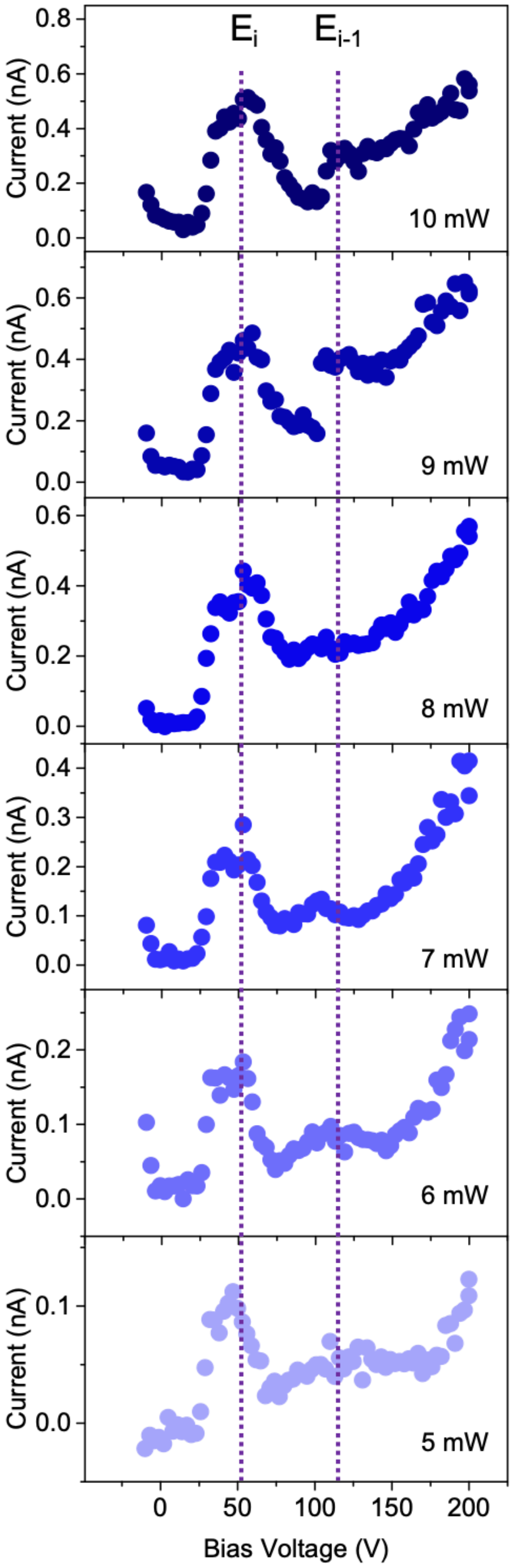

Fig. S3. I-V curves at different power from 5 mW to 10 mW at 20 K. Multiple peaks are observed.

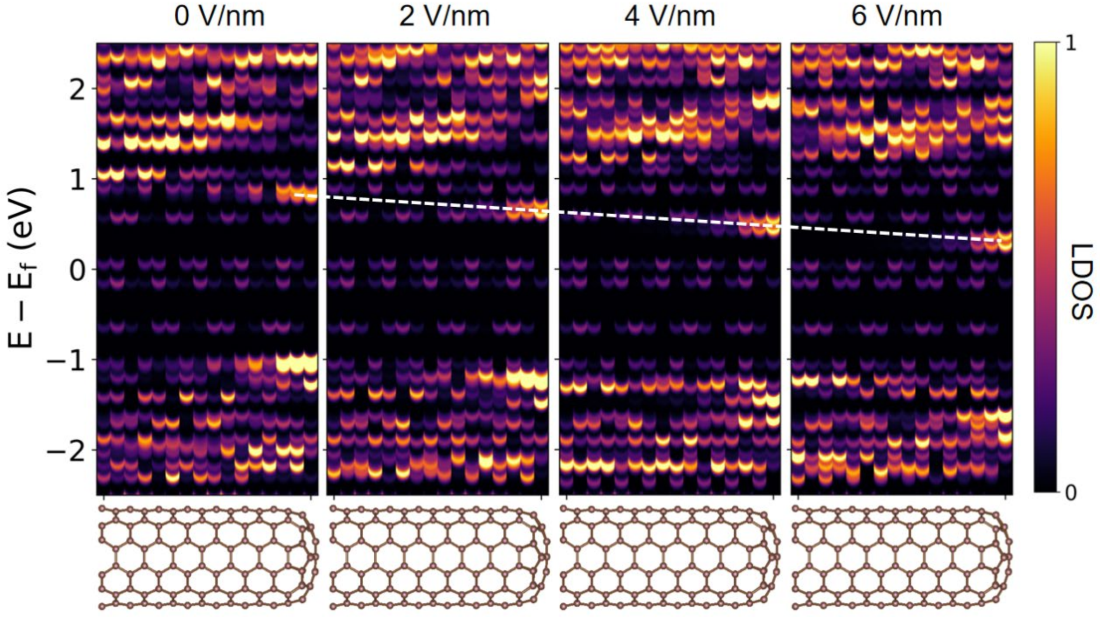

Fig. S4. Localized density of states (LDOS) of (6, 6) CNT under various static electric fields. The LDOS is averaged by summing carbon atoms with the same position along the axis of CNT. For the capped CNT, the tip is a semiconductor, while the body is a metal. Under an external static electric field, the localized electronic states originated from the apex undergo a clear shift around the Fermi level.

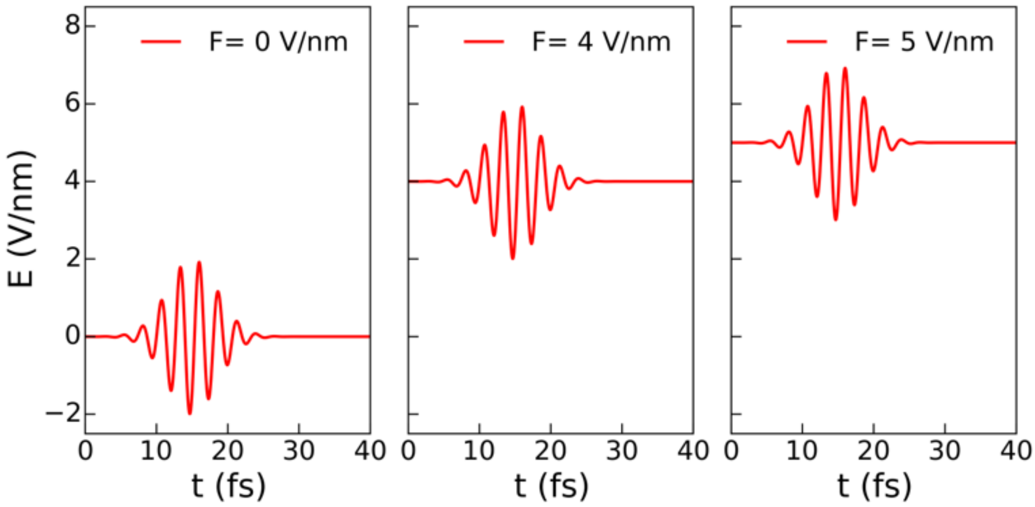

Fig. S5. Waveform of the external electric fields $\boldsymbol{E(t)}$ which are composed by a fixed ultrashort laser pulse and varied static electric fields. $E(t) = E_0 \cos(\omega t) \exp\left[-\frac{(t-t_0)^2}{2\sigma^2}\right] + F$. Here, F is the field strength of static (DC) field, the laser pulse reaches it maximum amplitude E_0=2 V/nm at t_0=15 fs and the full width at half maximum amplitude 2σ is 7 fs.

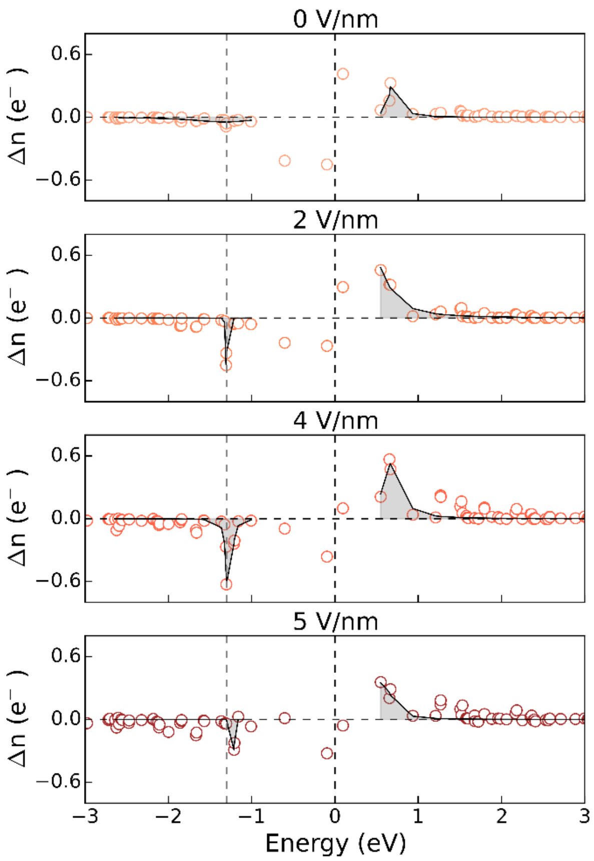

Fig. S6. Energy distribution of the excited carriers in (6, 6) CNT under the driven of four external fields. The grey shadowed areas are used to represent the dominant localized states that contribute to the carrier excitation.

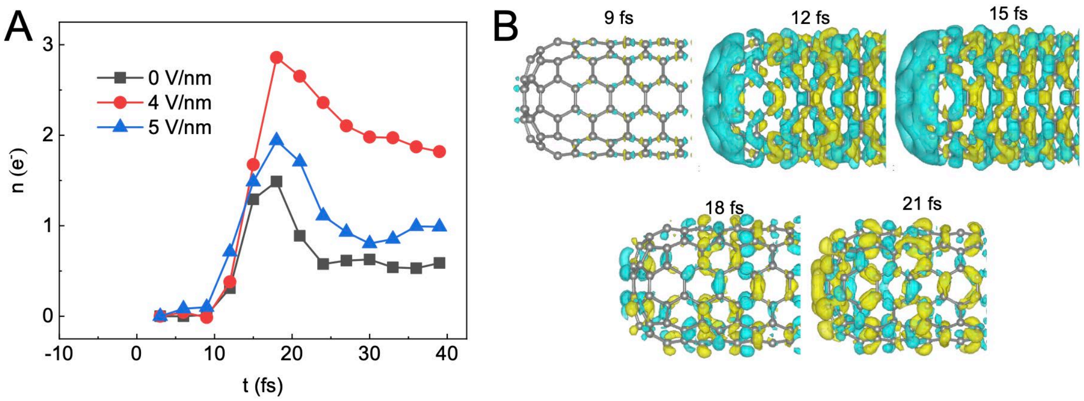

Fig. S7. Time-dependent carrier excitation and charge density distribution. (**A**) Time-evolution of number of excited electrons under the same laser field illumination but with varied static electric fields. (**B**) Time-resolved charge density differences of (6, 6) nanotube when static electric fields *F=0*. Here, the yellow and cyan colors represent the increase and decrease of charge density, respectively.

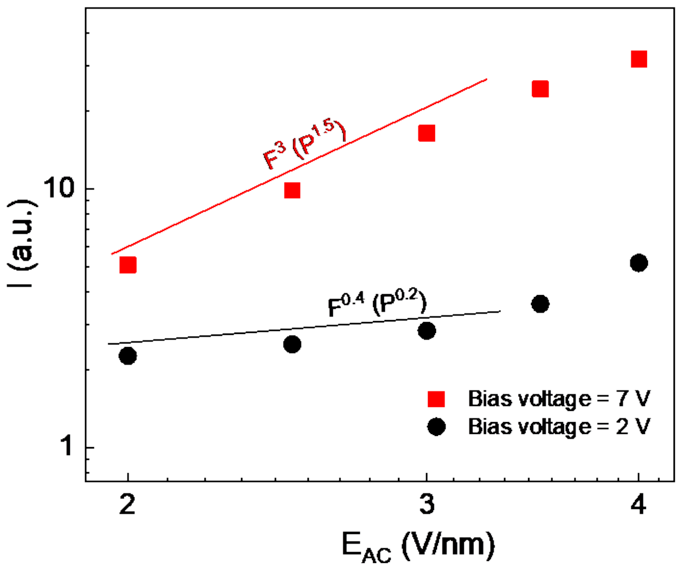

Fig. S8. Simulated emission current (I) as a function of laser field (F_{Laser}), at a bias voltage of 7V (corresponds to a static electric field of 3.5V/nm) and 2V (1V/nm).

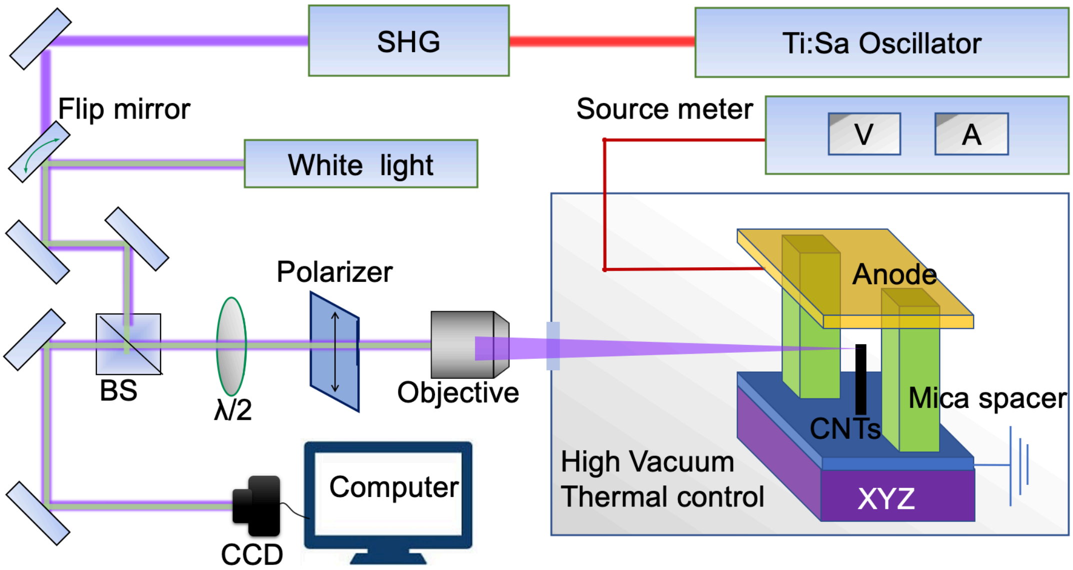

Fig. S9. Experimental setup. (SHG, second harmonic generation. CCD, charge-coupled device. BS, beam splitter.)

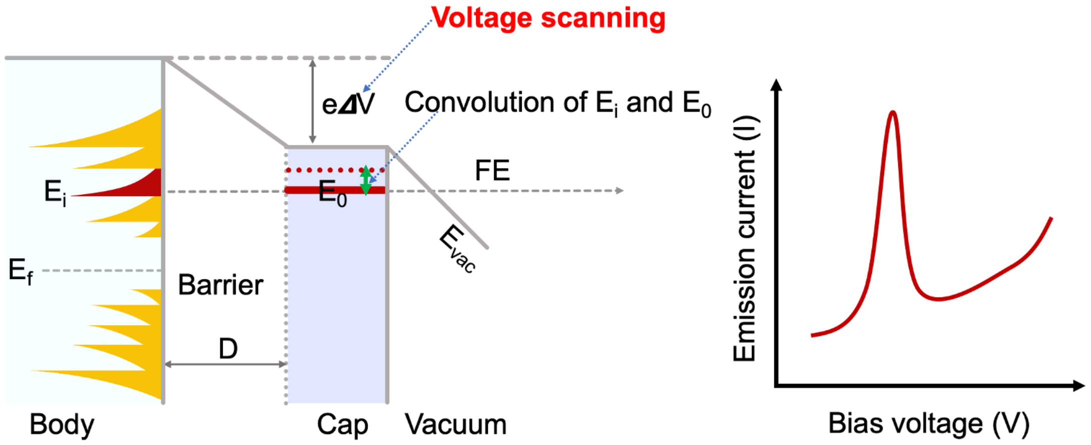

Fig. S10. Diagram of measurements of energy width. The energy width measurement in our work, which is the voltage scanning width that represents the convolution of E_i (an excited state) and E_0 (a quantized level in the cap).

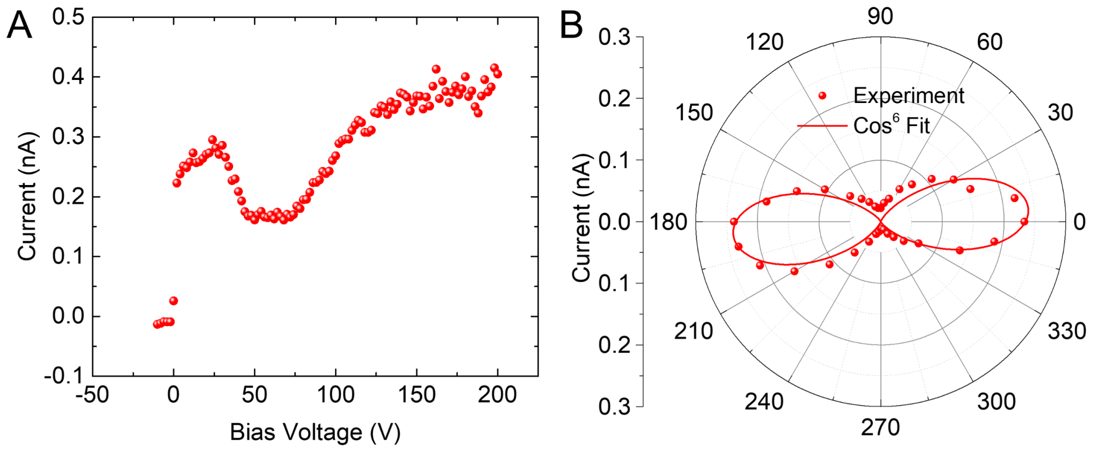

Fig. S11. Polarization dependent photoemission current. (**A**) I–V curves obtained at 78K, which shows NDR effect. (**B**) Experimentally obtained the emission current as a function of the laser polarization at a bias of 30 V. The solid line is the corresponding $\cos^6$ fit curve.

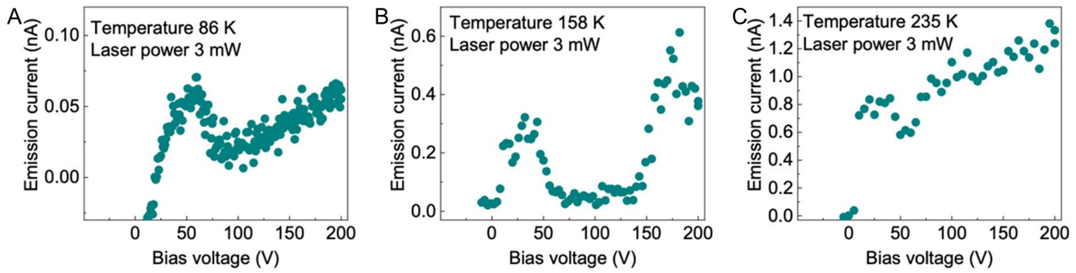

Fig. S12. Another group of temperature dependent data. Experimentally obtained voltage spacing of two adjacent NDR peaks as a function of temperature. (**A**)At lower temperature (86 K), only one NDR peak can be observed. (**B**) At moderate higher temperature (158 K), two NDR peaks can be observed with a clear gap. (**C**)At higher temperature (235 K) that approaching room temperature, the gap between the two peaks is almost invisible. Therefore, we can conclude that the gap between two NDR peaks is decreasing with the temperature increasing.

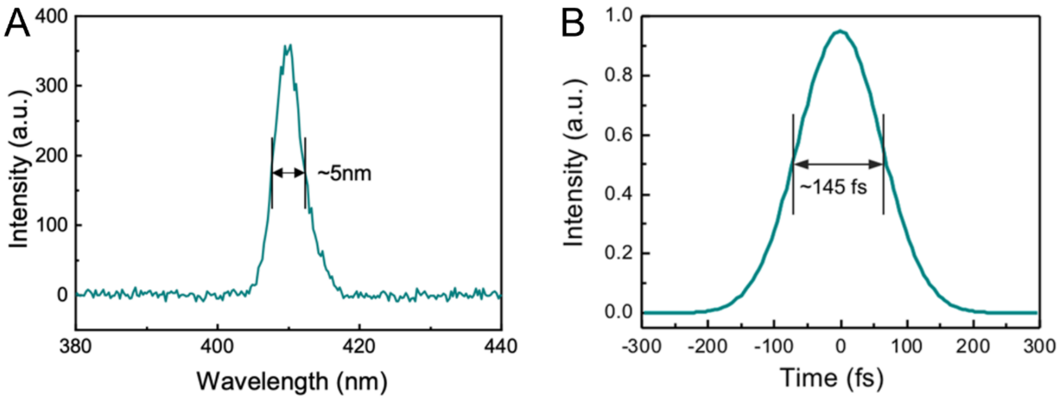

Fig. S13. The spectrum and the pulse duration of the 410nm laser. (**A**) The spectrum of the 410nm laser. (**B**) Autocorrelation measurement of 100-fs laser pulses. The autocorrelation function curve has a full-width-half-maximum (FWHM) of ~145 fs, which gives that the pulse width is $\frac{145\ \text{fs}}{\sqrt{2}} \approx 103\ \text{fs}$ (Gaussian function fitting). According to the statistical results of the many times of measured data, the pulse width is 103 ± 5 fs.

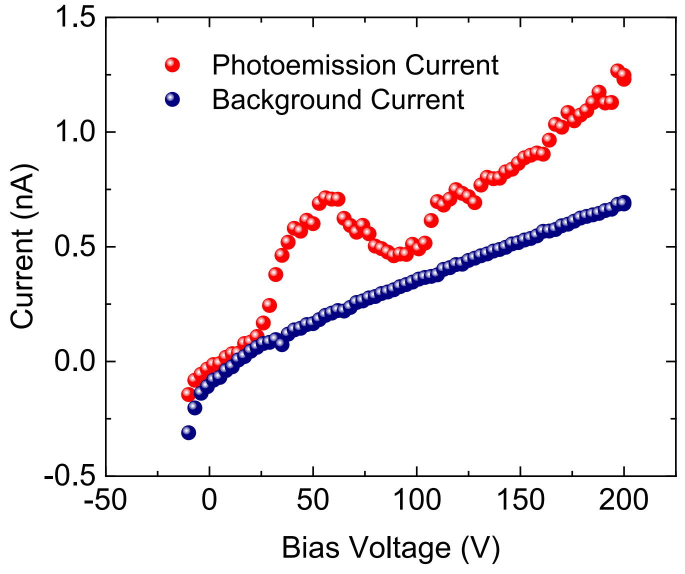

Fig. S14. **Comparison of photoemission current and background current.**

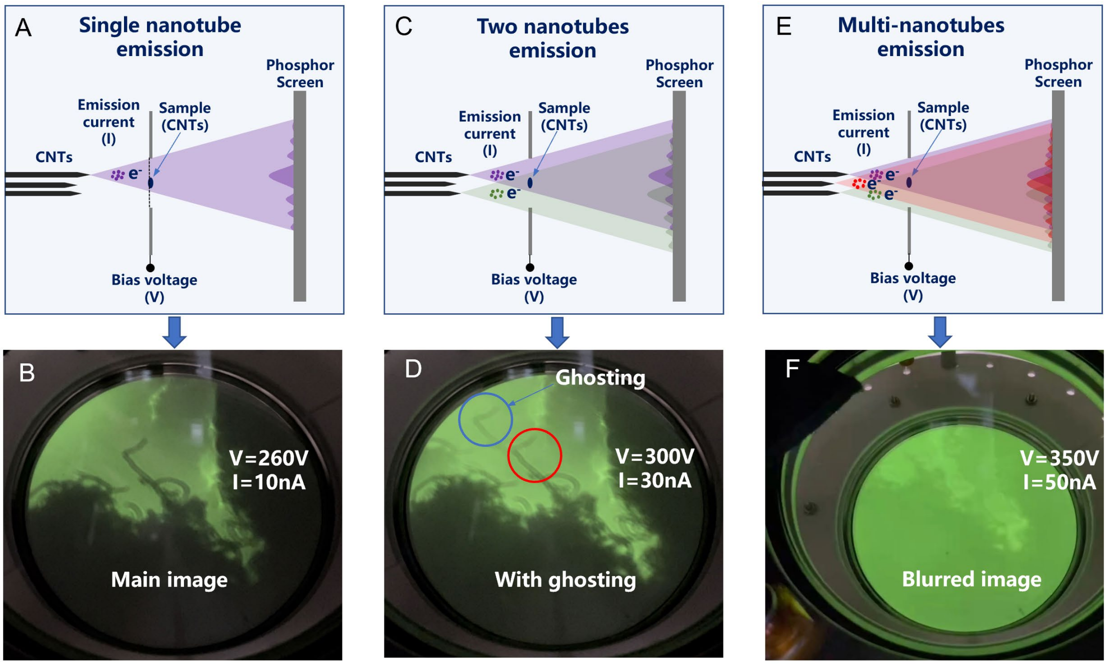

Fig. S15. Demonstration of single CNT emission at relatively low bias voltage. (**A**) shows the schematic diagram of PPM at relatively lower bias voltage and current (260V, 10nA)—single nanotube emission, while (**B**) shows the obtained microscopy image. (**C**) shows the schematic diagram of PPM at a slightly higher bias voltage and current (300V, 30nA)—two nanotubes emission, while (**D**) shows the obtained image with ghosting. (**E**) shows the schematic diagram of PPM at a higher bias voltage and current (350V, 50nA)—multiple nanotubes emission, while (**F**) shows the obtained blurred image.

Data file S1. Data and code for the manuscript.
