# Supplementary figures and images for: Coherent ultrafast photoemission from a single quantized state of a one-dimensional emitter
Source: Sci Adv. 2023 Oct 12;9(41):eadf4170. doi: 10.1126/sciadv.adf4170 (PMC10569710; doi:10.1126/sciadv.adf4170)

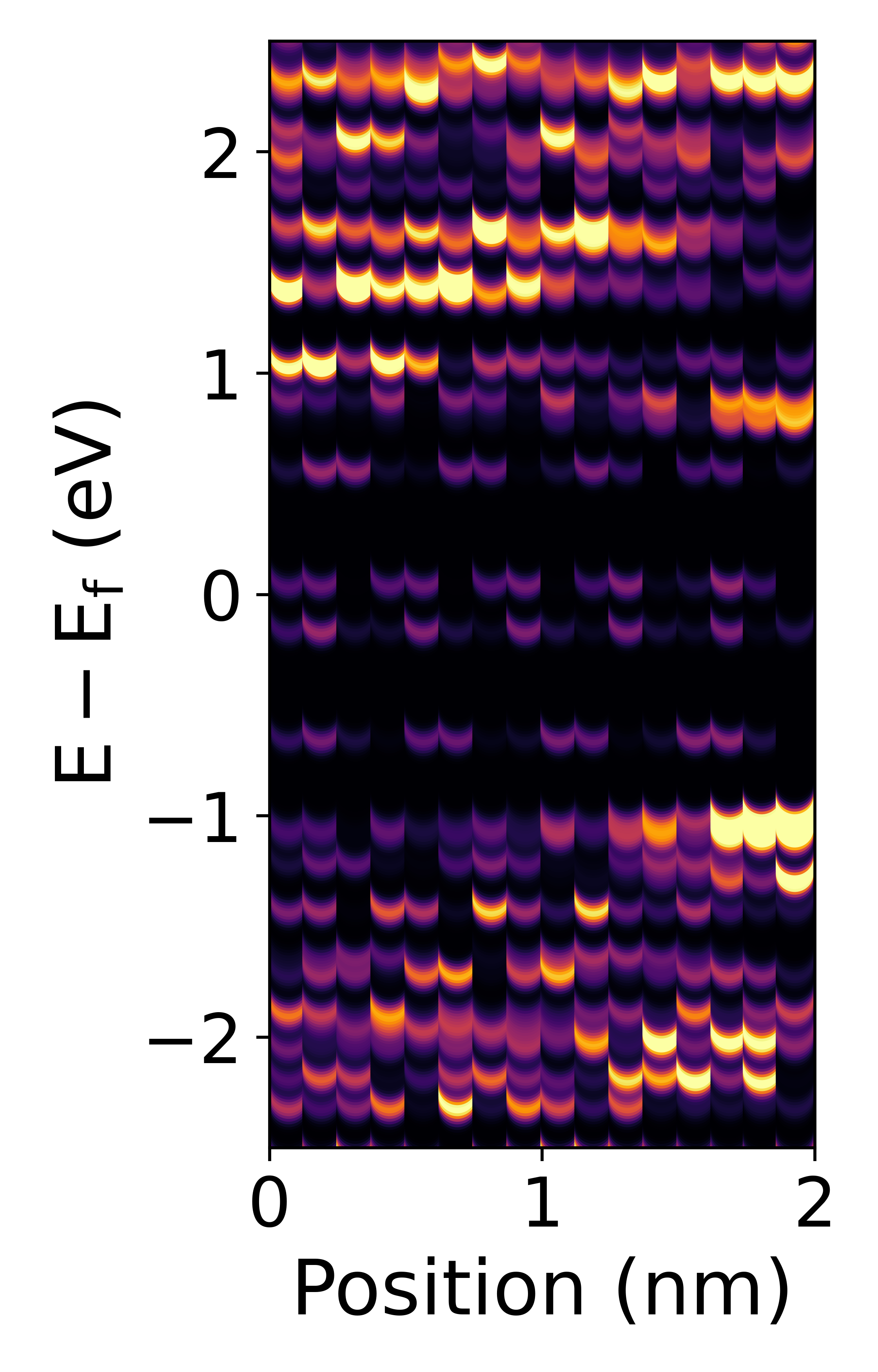

Supplement: Supplementary file 2 — Data file S1 [file sciadv.adf4170_data_file_s1.zip › data and code/data_figure_4/Figure4B/0fs.png]

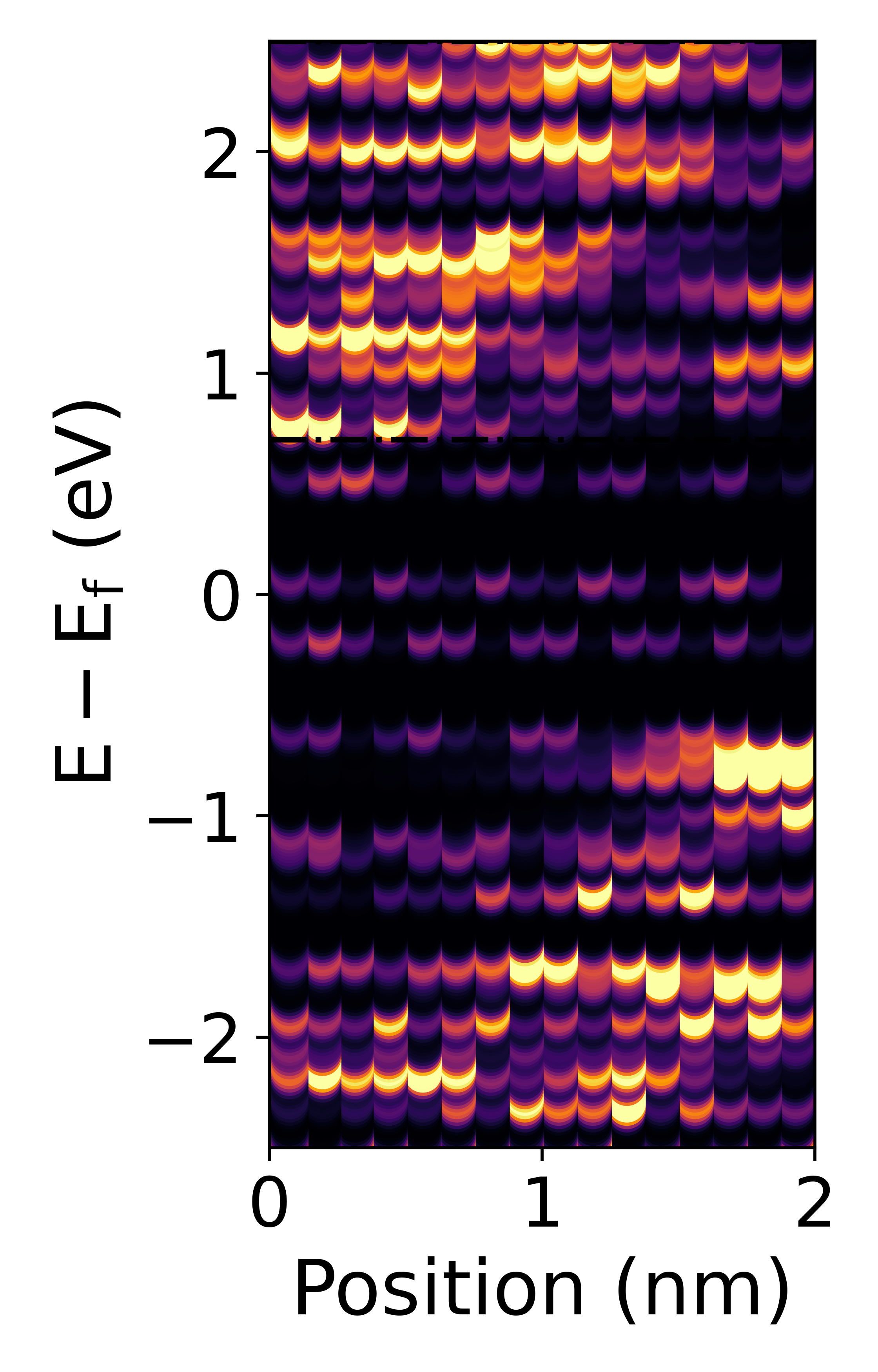

Supplement: Supplementary file 2 — Data file S1 [file sciadv.adf4170_data_file_s1.zip › data and code/data_figure_4/Figure4B/15fs_test.png]

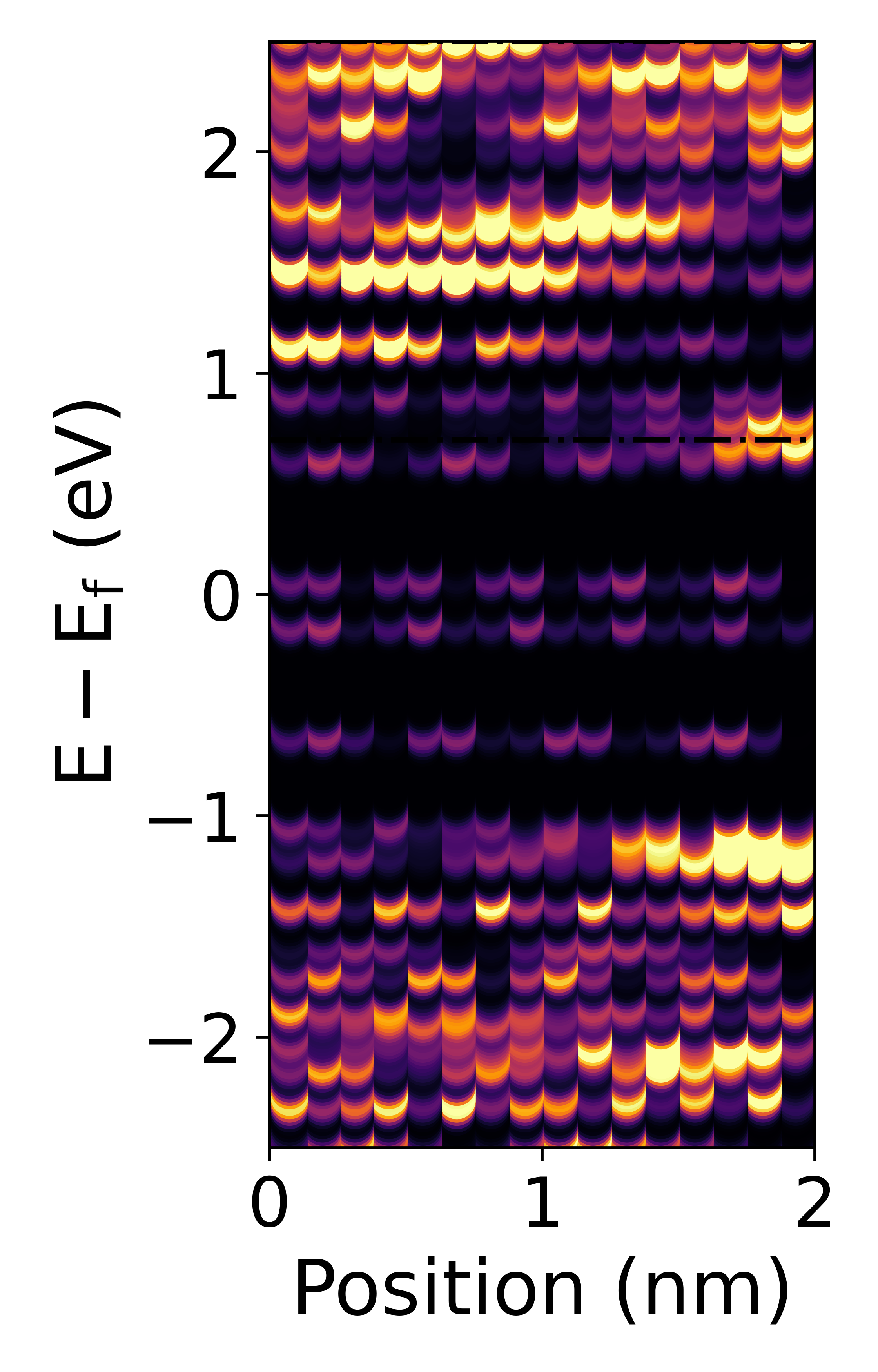

Supplement: Supplementary file 2 — Data file S1 [file sciadv.adf4170_data_file_s1.zip › data and code/data_figure_4/Figure4B/27fs_test.png]

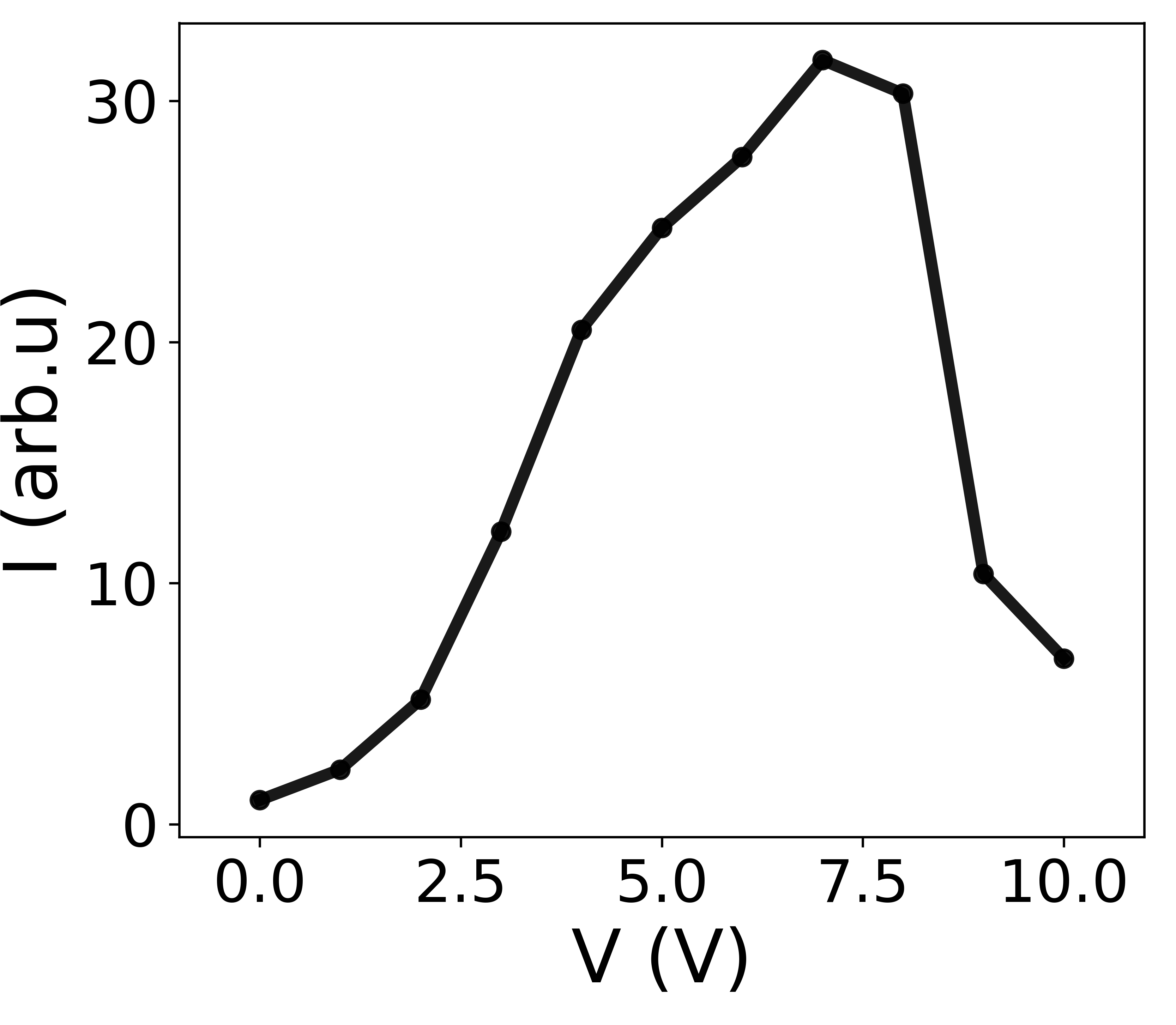

Supplement: Supplementary file 2 — Data file S1 [file sciadv.adf4170_data_file_s1.zip › data and code/data_figure_4/Figure4C/total_excited_e2 - ╕▒▒╛.png]

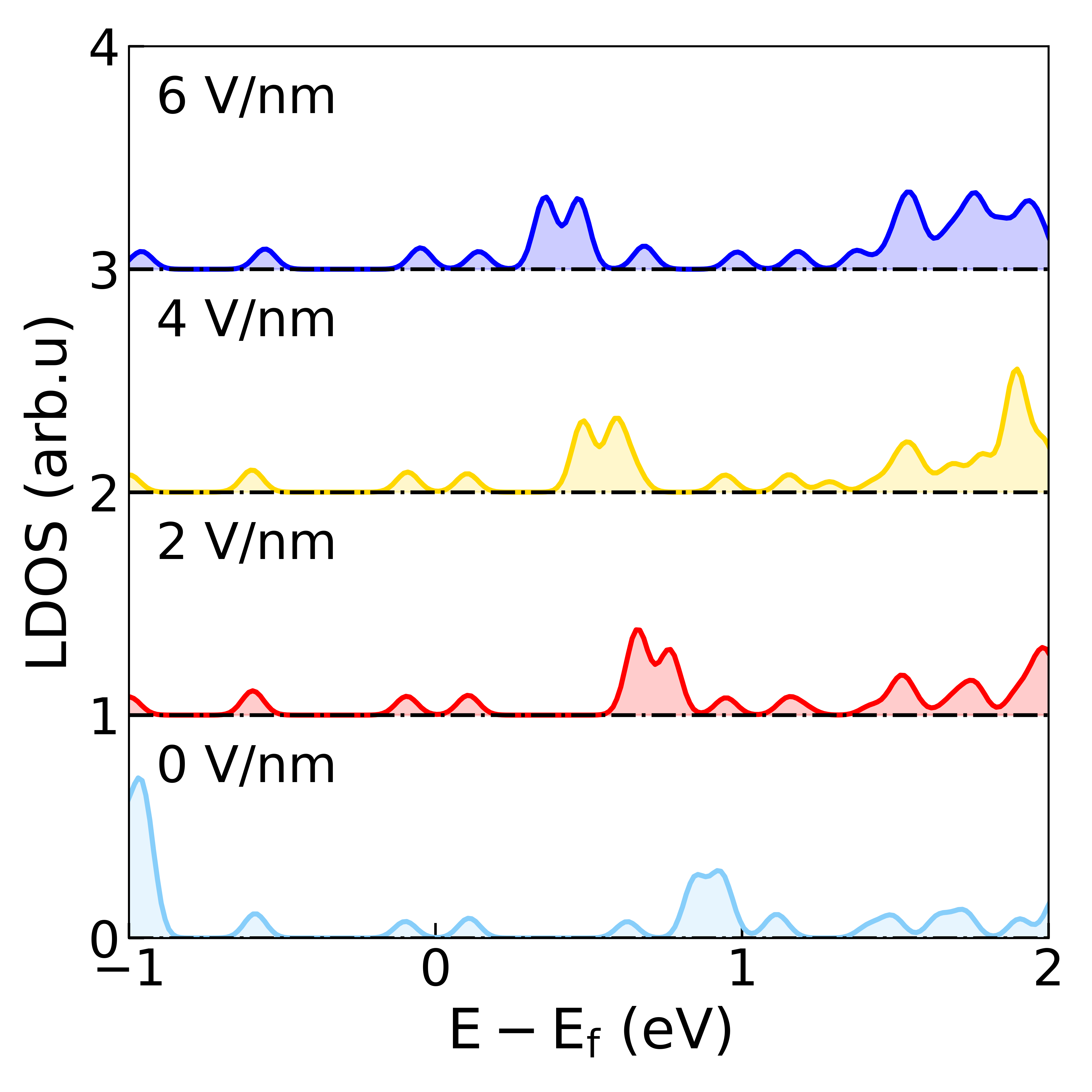

Supplement: Supplementary file 2 — Data file S1 [file sciadv.adf4170_data_file_s1.zip › data and code/data_figure_4/Figure4D/inset/cap_testpeak_0246.png]
